# Preparation of PNT@SiO_2_ Aerogel Composite Phase Change Material with Oriented Structure and Its Thermal Management Characteristics for Battery

**DOI:** 10.3390/nano16120709

**Published:** 2026-06-09

**Authors:** Silong Wang, Wei Yan, Pan Sun, Jun Yuan

**Affiliations:** School of Nuclear Science, Energy and Power Engineering, Shandong University, Jinan 250061, China

**Keywords:** composite phase change materials, power battery thermal management, oriented structure, aerogel

## Abstract

Power batteries used in electric-powered vessels, new-energy tractors or construction machinery typically require prolonged, continuous operation at high power levels, which can lead to significant heat buildup and pose serious threats to battery safety, cycle life, and operational stability. Traditional air-cooled and liquid-cooled systems struggle to meet the requirements for efficient heat dissipation under heavy loads. Phase change materials (PCMs) are ideal for passive battery thermal management due to their high latent heat but are severely limited by low thermal conductivity and liquid leakage. In this study, nitrogen-doped carbon nanotubes@SiO_2_ (PNT@SiO_2_) were synthesized and further fabricated into oriented porous aerogels by directional freeze-drying using cellulose-based materials as the skeleton. Polyethylene glycol-8000 (PEG-8000) was loaded via vacuum impregnation to obtain the PSAP composite PCM. The optimized composite exhibits a thermal conductivity of 0.93 W/m·K, 3.2 times that of pure PEG, with 96% PEG loading and a phase change enthalpy of 158 J/g. Battery thermal management tests demonstrate its excellent temperature control and heat suppression performance. This study provides a high-performance and feasible thermal management solution for power batteries used in relevant fields.

## 1. Introduction

Electric-powered vessels, new-energy tractors and construction machinery operate in extremely harsh environments and must run continuously at high power levels [1]. The demand for high power has driven a continuous increase in battery energy density; the resulting heat buildup can cause the battery temperature to rise excessively, leading to irreversible structural degradation, significantly shortening its service life, and even posing a risk of thermal runaway [2]. Against this backdrop, effective thermal management of power batteries is a key technology for ensuring their rapid development [3].

According to Ohm’s law and the law of conservation of energy, in addition to battery performance degradation and life extension, the large amount of heat generated by the power battery will also lead to significant electrical energy loss, thereby imposing additional load on the entire engineering equipment power system. Severe heat accumulation in power batteries not only poses significant safety risks but also leads to substantial energy waste, which has become a key constraint on the further application and industrial development of power batteries. Accordingly, developing high-efficiency thermal management technology for power batteries is of great practical significance and an urgent necessity [4,5].

Currently, the mainstream cooling methods for power battery systems are primarily air cooling and liquid cooling. Although both cooling methods are effective for thermal management, the addition of phase change materials can help balance battery temperatures and improve thermal management performance [6]. The high latent heat of PCMs makes them promising candidates for thermal management, and an increasing number of researchers are focusing on phase change cooling [7,8,9]. PCM’s low thermal conductivity and poor shape stability during phase change necessitate incorporating a small amount of an efficient thermal-conducting filler and a shape-stable skeleton to prepare a composite PCM for thermal management applications in power battery systems [10,11].

Many related studies have reported various preparation methods and guidance for the application of composite PCMs. Among them, aerogel with strong adsorption, stable structure, and good material compatibility has become an important base material for preparing composite phase change materials. The porous microstructure of aerogel materials provides a capillary effect for PCM in phase change, so that liquid PCM will not leak [12]. Aerogels typically consist of a continuous network of high-thermal-conductivity fillers (e.g., carbon nanotubes and graphene) and shape-stable matrix materials (e.g., cellulose and polyethyleneimine) that interconnect the fillers and maintain structural integrity. Using such components, numerous aerogel-based composite PCMs have been developed with enhanced thermal conductivity and shape stability, enabling excellent thermal management performance [13,14].

Aerogel-supported composite PCMs have become a research focus in high-performance thermal management, and the synergistic design of carbon nanomaterials, polymer matrices, and preparation processes is crucial to balancing thermal conductivity and latent heat. In the development of PVA-based composite systems, the incorporation of CNTs and graphene has been widely adopted to enhance heat-transfer performance. Yet differences in dispersion state, structural orientation, and interfacial construction lead to significant discrepancies in final properties. Traditional blending and non-directional freeze-drying methods can only achieve random dispersion of thermally conductive fillers, failing to form continuous and effective heat-conduction paths; even with the support of PVA aerogel and a high phase change enthalpy, thermal conductivity remains limited to around 0.51–0.56 W/m·K [15,16]. Although directional freezing combined with ultra-high temperature graphitization can significantly optimize the ordered arrangement of graphene and repair structural defects, thereby constructing a continuous anisotropic thermal conduction network and increasing thermal conductivity up to 1.37 W/m·K while maintaining high latent heat, such strategies rely on harsh preparation conditions and high energy consumption, which restrict large-scale preparation and application. In contrast, external coating modification can achieve a relatively mild preparation process. Still, it is difficult to form tight, coherent heat-conducting paths between the outer coating and the internal aerogel matrix, resulting in limited improvement in thermal conductivity [17]. Taken together, the above systems show that simply adding fillers, directional orientation under extreme conditions, or passive external modification cannot fully meet the requirements of high efficiency, stability, and low-cost preparation. Therefore, the core path to further improve the thermal management performance of composite phase change materials is to build a dense, continuous, and orderly thermal conduction network under appropriate preparation conditions, thereby achieving high thermal conductivity, high latent heat of phase change, and excellent structural stability simultaneously.

To address the demands for thermal safety and high-efficiency thermal management in power batteries, this study successfully developed composite phase change materials featuring high thermal conductivity, large phase change enthalpy, excellent shape stability, and outstanding thermal management performance. PNT@SiO_2_ were employed as a thermal conductivity enhancer, while a cellulose-based-oriented aerogel skeleton and a PEG phase change matrix were synergistically integrated. Through material design, controllable preparation, microstructure characterization, and thermal performance test of the system, the structure–activity relationship among nano-morphology regulation, aerogel skeleton construction, and phase change behavior was fully revealed, which provided experimental basis and theoretical support for the application of aerogel-based composite phase change materials in the field of battery thermal safety.

## 2. Materials and Methods

### 2.1. Materials

Pyrrole (AR) was purchased from Anhui Senrise Technologies Co., Ltd, Anqing, China. FeCl_3_·6H_2_O (AR), Methyl orange (AR) and Tetraethyl orthosilicate (TEOS) (98%) were all purchased from Shanghai Macklin Biochemical Co., Ltd, Shanghai, China. Ammonia water (AR) was purchased from Yantai Yuandong Fine Chemicals Co., Ltd, Yantai, China. PEG-8000 (Mn = 8000 g/mol, 99 wt%) was purchased from Beijing Mreda Technology Co., Ltd, Beijing, China. Absolute ethyl alcohol (98 wt%) was purchased from Tianjin Fuyu Fine Chemical Co., Ltd, Tianjin, China. cellulose nanCellulose nanofibers (CNFs) (3 wt% in water) was purchased from Saoxing Yuewei Advanced Materials Co., Ltd, Shaoxing, China. Sodium carboxymethyl cellulose (CMC) (viscosity: 600–300 mpa.s, USP grade) was purchased from Hangzhou Yanqu Information Technology Co., Ltd, Hangzhou, China. All reagents were used directly without further purification.

### 2.2. Preparation of PNT and PS

Polypyrrole (PPy) tubes were prepared by the classical one-pot oxidation method with FeCl_3_, Methyl orange and Pyrrole [18]. PNT was fabricated by pyrolyzing PPy in a nitrogen atmosphere.

Preparation of SiO_2_ nanospheres by the Stöber method [19]. Add 1 g of 1 g PNT powder into 100 mL of absolute ethanol, fully stir and ultrasonically treat the suspension in an ultrasonic cell crusher for 1 h; then, add 2 mL of deionized water, 1 mL of ammonia water and 2.5 mL TEOS, react at room temperature for about 12 h, add 1 mL TEOS for the second time, continue the reaction for about 12 h, add 1 mL TEOS for the third time, add 1 mL of TEOS for the fourth time, and stop the reaction for 12 h. Remove the mixed suspension; then, centrifugally wash it with deionized water at 6000 r/min for 3 times, with anhydrous ethanol at 3000 r/min for 1 time, and dry the obtained precipitate at 60 °C for 6 h to obtain PNT@SiO_2_ (PS).

### 2.3. Preparation of Aerogel Composite PCMs

**Figure 1:**
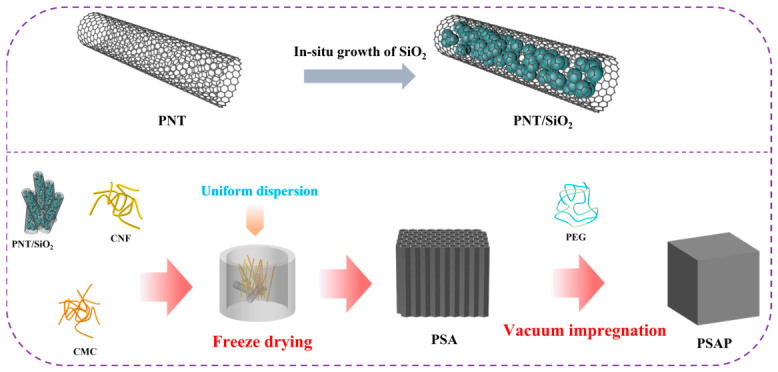
Preparation processes of PS and PSAP.

Put 1 g PS powder into 200 mL deionized water, perform ultrasonic treatment as PNT, add 1 g CMC powder, stir at 500 r/min for 2 h; then, add 30 g CNF, stir at a high speed of 1200 r/min for 12 h, and after stirring evenly, transfer the obtained viscous suspension to a mold with copper as the bottom material and PTFE as the wall material, with the size of 18,650 commercial lithium battery as the standard. Freeze in a low-temperature refrigerator at −20 °C for 12 h, vacuum freeze-dry in a freeze dryer at −80 °C for 36 h to obtain PS aerogels, replace 1 g PNT powder with 1 g PS powder, and obtain PNT aerogels under the same other conditions. The two aerogels were named PSA (PNT@SiO_2_ aerogel) and PA (PNT aerogel), respectively. The preparation process is shown in Figure 1.

PSA and excess PEG were placed in a container and vacuum-impregnated in a vacuum oven at 80 °C for 24 h. After being taken out, the sample was placed on filter paper, and the excess PEG was leached in a blast oven at 80 °C. The final aerogel composite PCM was named PSAP, PA replaced PSA, and other conditions were unchanged, so the aerogel composite PCM was obtained and named PAP.

### 2.4. Characterization Testing Technology and Related Instruments

See Supporting Information for test and characterization methods and technical details.

## 3. Results and Discussion

### 3.1. Microstructure Characterization of Aerogel Composite PCMs

The microstructural morphology of as-prepared PA and PSAs was characterized by SEM and TEM to reveal the morphological difference and structural evolution after SiO_2_ modification.

**Figure 2:**
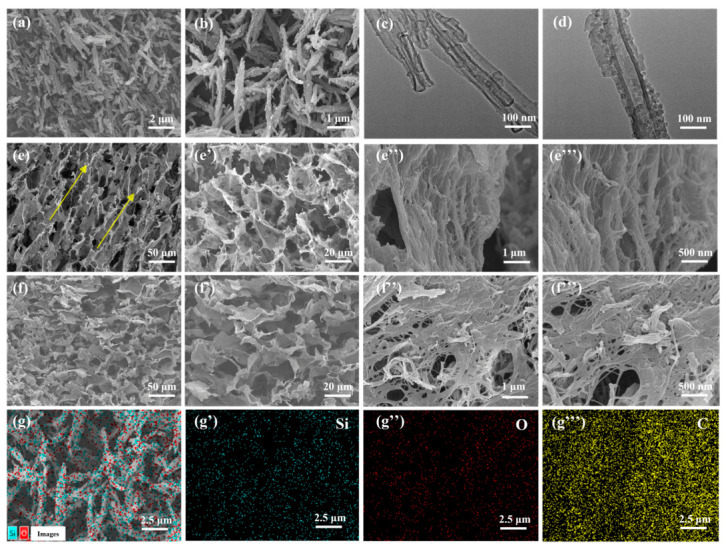
(**a**) SEM images of PNT; (**b**) SEM images of PNT@SiO_2_; (**c**) TEM images of PNT; (**d**) TEM image of the PNT@SiO_2_; (**e**–**e‴**) SEM image of PSA; (**f**–**f‴**) SEM image of the PA; (**g**–**g‴**) local surface SEM image of PNT@SiO_2_, along with the EDS elemental mapping of Si, O and C.

As shown in Figure 2a–d, PNT and PNT@SiO_2_ (PS) exhibit similar overall tubular morphology, while obvious differences can be observed at the microscale. A small number of SiO_2_ nanospheres are dispersed on the outer surface of PNT, and more nanoparticles are uniformly embedded inside the tubular cavity of PNT. The dense distribution of SiO_2_ nanospheres can form additional thermal conduction nodes and continuous heat-transfer pathways, which is expected to optimize the interfacial thermal contact between pristine carbon nanotubes and improve thermal conduction efficiency. Figure 2d was analyzed by the ImagJ 1.x software, and the average diameter of SiO_2_ rice balls on PS was 28.2 nm.

Figure 2e,f compares the microstructures of PSA and PAs. Benefiting from directional freeze-drying and the high thermal conductivity of PS filler, PSA presents a well-defined, oriented, and aligned porous structure along the ice crystal growth direction. In contrast, PA exhibits a relatively disordered, loosely stacked structure. Such a morphological distinction is mainly attributed to the difference in thermal conductivity and interfacial compatibility between PNT and PS particles during the freezing self-assembly process. Furthermore, all aerogel samples possess hierarchical pore characteristics, including micron-scale macropores and nano-scale pores formed by the interconnection of PNT and CNF networks. The multistage porous structure provides sufficient adsorption space and a capillary confinement effect, which are essential for high-loading encapsulation of PEG and for leakage suppression during the solid–liquid phase transition.

### 3.2. Structure Analysis of Aerogel Composite PCMs

**Figure 3:**
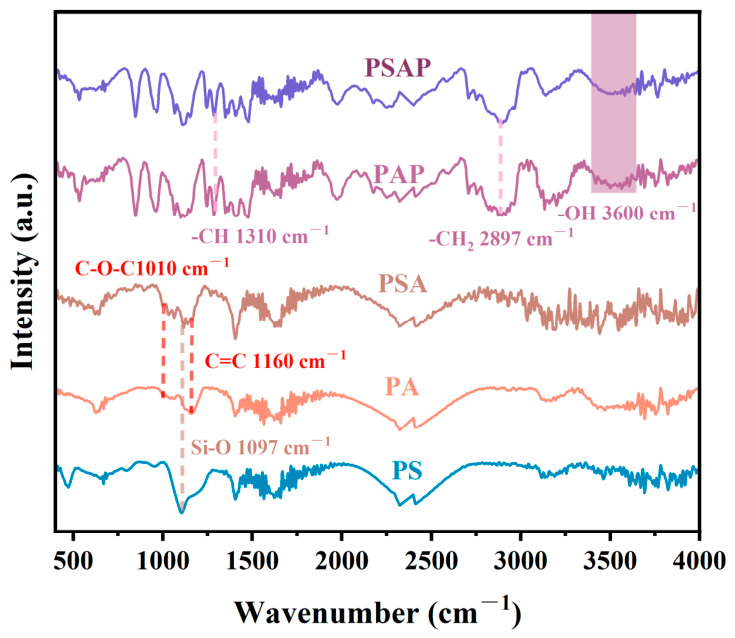
The FT-IR spectra of PS, PA, PSA, PAP, and PSAP, respectively.

FT-IR spectroscopy was employed to analyze the chemical composition and intermolecular interaction of PS, PA, PSA, PAP, and PSAP, as displayed in Figure 3. The characteristic absorption peak at 1097 cm^−1^ assigned to the Si–O stretching vibration is clearly detected in PS and PSA spectra, verifying the successful fabrication of SiO_2_ and stable combination with PNT in the aerogel skeleton [20]. The typical characteristic peaks of PEG at 1310 cm^−1^, 2897 cm^−1^, and 3600 cm^−1^ correspond to the stretching vibration of –CH–, –CH_2_– [21] and –OH groups [22], respectively. These characteristic signals are well retained in PSAP and PAP without obvious shift or attenuation, demonstrating that the impregnation of PEG does not damage the intrinsic chemical structure of the cellulose aerogel skeleton and PNT@SiO_2_ filler. Meanwhile, the characteristic peaks of CNF (C–O–C at 1010 cm^−1^ and C=C at 1160 cm^−1^) can be identified in PA and PSA, further confirming the successful construction of the cellulose-based supporting network [23]. Due to the equipment’s long service life, some noise interference has appeared in the raw data; to avoid affecting the analysis results, no excessive smoothing was applied.

**Figure 4:**
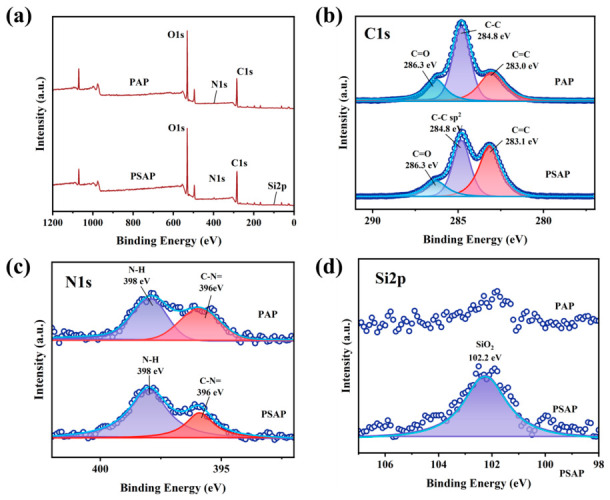
(**a**) XPS survey spectra of PSAP and PAP, XPS spectra of PSAP and PAP, for (**b**) C1s, (**c**) N1s and (**d**) Si2p.

XPS was used to assess elemental composition and chemical state differences between PAP and PSAP surfaces, and the results are shown in Figure 4. From Figure 4a,b, i.e., the XPS high-resolution full spectrum and the Si2p XPS spectrum of PSAP and PAP [24], it can be seen that PAP has lower Si content than PSAP, which is due to the difference in SiO_2_ content between them. From Figure 4b,c, that is, the C1S XPS spectrum and the N1S XPS spectrum, we can see that the C–C (284.8 eV), C=C (283.1 eV), and C=O (286.3 eV) [25], pyrrole nitrogen (396 eV) and N–H (398 eV) [26] brought by PNT are in. The above results show that the components are combined primarily through physical entanglement and hydrogen bonding rather than chemical reaction, which is favorable for maintaining the respective functional properties of each constituent.

### 3.3. Thermal Management Performance of Aerogel Composite PCMs

**Figure 5:**
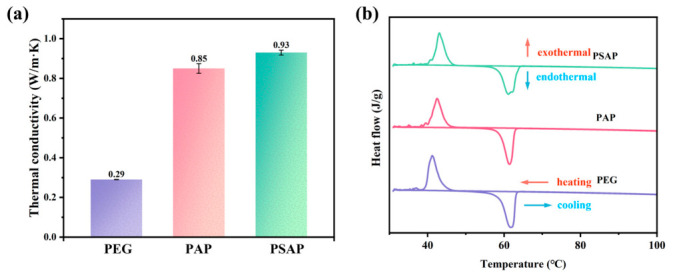
(**a**) Thermal conductivity of PEG, PAP, and PSAP, respectively; (**b**) the DSC curves of PEG, PAP, and PSAP, respectively.

Figure 5a shows the thermal conductivity of PEG, PAP, and PSAP. Therefore, the thermal conductivity of PSAP can reach 0.93 W/m·K, which is 3.2 times that of PEG (0.29 W/m·K), and that of PA can also reach 2.93 times that of PEG. There may be two reasons for this obvious difference: one is the existence of PNT. As a nitrogen-doped carbon nanotube material, PNT possesses intrinsic high thermal conductivity, and its incorporation can effectively enhance the heat-conducting performance of composite systems [27,28]. Moreover, owing to the adopted directional freeze-drying strategy, the resulting aerogel-based composite PCM exhibits anisotropic thermal conductivity, with superior heat-transfer capability along the freezing direction, namely, the ice-crystal growth orientation. Aerogels fabricated via this method can form continuous, interconnected thermal-conducting pathways throughout the entire material framework [29].

Figure 5b shows the phase-transition characteristics of PEG, PAP, and PSAP as shown by DSC curves. The phase-transition enthalpy of an aerogel composite PCM is often determined by its PEG content. According to the quality comparison of PA, PSA, PAP, and PSAP, the PEG loading rates of PAP and PSAP are 95% and 96%, respectively. Such a high loading rate often stems from the special porous structure of aerogels, and the higher PSAP than PAP may be due to PS having a stronger adsorption capacity than PNT. The phase transition enthalpies of PAP and PSAP reach 154.1 J/g and 158 J/g, respectively, which are 10.15% and 7.87% lower than those of PEG, consistent with the loading of PEG into PAP and PSAP. The specific phase change properties are shown in Table 1. PAP and PSAP have high thermal conductivity and high phase-transition enthalpy, which make them theoretically capable of thermal management.

**Figure 6:**
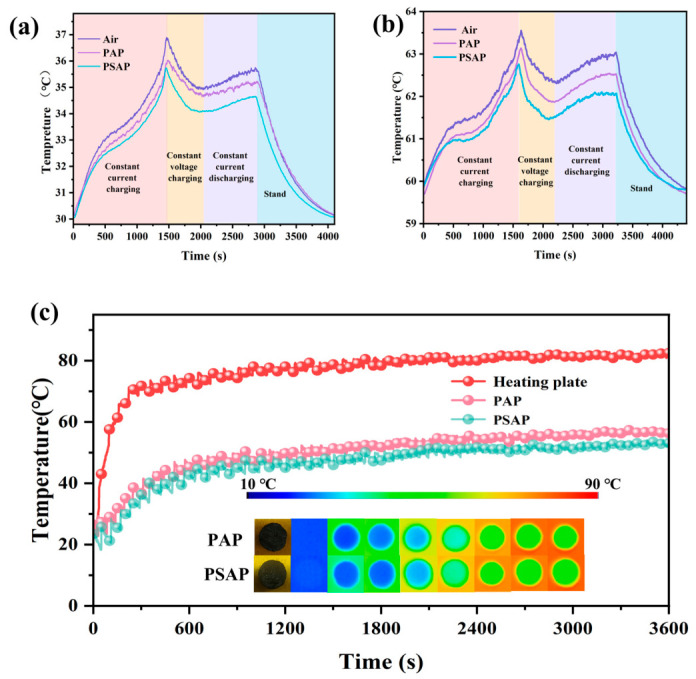
Surface temperature of battery at 30 °C (**a**) and 60 °C (**b**); (**c**) time–temperature curve of the heating plate, PAP and PSAP at different times.

Based on thermal conductivity and DSC results, both PAP and PSAP exhibit promising potential for comprehensive thermal regulation. To objectively evaluate their practical battery thermal management performance, multiple repeated charge–discharge tests were carried out on 18,650 lithium-ion cells encapsulated with tailored PAP and PSAP samples. All experiments were implemented at a 2C rate under ambient temperatures of 30 °C and 60 °C, and real-time surface temperature evolution was recorded using high-sensitivity thermocouples, as summarized in Figure 6a,b.

Test results show that both samples effectively suppress the rise in battery temperature under different operating conditions. At 30 °C, PSAP reduces the battery surface temperature by 1.5 °C, outperforming PAP, which reduces it by 1.0 °C. When the ambient temperature increases to 60 °C, the corresponding temperature decrease declines slightly to 1.3 °C and 0.6 °C for PSAP and PAP, respectively. Although the absolute temperature drop of 0.6–1.5 K seems relatively moderate at first glance, this phenomenon can be reasonably explained from the following three aspects: inherent battery heat generation, coating thickness, and material design positioning. First, although the test was performed at a 2C rate, the selected 18,650 cell exhibits an intrinsically low heat generation rate. The peak surface temperature reaches only 63.6 °C at 1500 s during charging, indicating a low baseline temperature rise and a gentle heating rate. With good thermal-response sensitivity, PAP and PSAP can simultaneously buffer temperature variations; thus, the macroscopic temperature difference is naturally limited under mild battery heat generation. Second, the as-prepared composite adopts an ultra-thin conformal wrapping design, with the actual coating thickness of PSAP only 1.9 mm. As confirmed in the previous literature [30], a thinner PCM coating inevitably leads to a limited temperature-suppression effect, whereas most reported systems with a more pronounced cooling effect usually employ much thicker PCM layers or large-volume PCM blocks to provide sufficient heat storage capacity. Third, this work prioritizes the lightweight and compact packaging requirements of power batteries for engineering machinery. The coating thickness and overall material volume are deliberately controlled, rather than pursuing an exaggerated temperature reduction by increasing material dosage. The slight attenuation in thermal regulation performance at 60 °C is also attributed to the battery surface temperature gradually approaching PEG’s phase-transition temperature, thereby reducing the latent-heat absorption margin and weakening the instantaneous cooling capacity. Even so, PSAP maintains a stable, reliable temperature-buffering capability. In addition, Table 2 compares the performance of composite materials reported in the literature [31,32,33,34] with the comprehensive thermal properties of the PSAP prepared in this study.

A further hot-plate test at 80 °C was conducted to simulate harsh long-term service conditions and verify the passive thermal stabilization performance; the temperature curves are presented in Figure 6c. After long-term operation, the surface temperatures of PAP and PSAP gradually stabilize at 57 °C and 52 °C, respectively. Such a high-temperature, constant working condition is more consistent with the actual service environment of engineering machinery power batteries. Even under the dual constraints of low intrinsic battery heat generation and an ultra-thin, lightweight structure, PSAP can still effectively restrain continuous temperature rise, smooth thermal fluctuations, alleviate heat accumulation, and delay battery aging, demonstrating important practical engineering value for long-term high-load battery operation. Subsequent analysis will further compare this work with recently reported lightweight thin-coating PCM thermal management schemes to clarify the comprehensive advantages and application positioning of the as-prepared materials.

## 4. Conclusions

With carboxymethyl cellulose (CMC) and cellulose nanofibers (CNFs) employed as the flexible supporting skeleton, PNT-based aerogels (PAs) and PNT@SiO_2_-based aerogels (PSAs) were fabricated by directional freeze-drying. Both aerogels present an oriented porous structure aligned along the ice crystal growth direction, as well as a multistage channel system comprising micron-scale macropores and nanoscale mesopores. Such structural features endow the aerogels with superior mechanical stability and shape-retention capability, while providing sufficient adsorption space and capillary forces for polyethylene glycol (PEG). SiO_2_ modification optimizes the surface energy and hydrophobicity of PSA, resulting in a high PEG adsorption rate of 96%, exceeding that of PA (95%).

FT-IR and XPS results demonstrated that only physical assembly and hydrogen bonding exist among PNT, SiO_2_, cellulose skeleton, and PEG, without any chemical reaction. Hence, the intrinsic phase change properties of PEG and the functional characteristics of each component are well-preserved.

Thermal performance tests indicated that pure PEG exhibits a low thermal conductivity of 0.29 W/m·K. By contrast, PAP reaches 0.85 W/m·K, and PSAP further increases to 0.93 W/m·K, which is 3.2 times that of pure PEG. This confirms that the synergistic effect of PNT@SiO_2_ and the oriented skeleton yields a highly efficient, continuous three-dimensional thermal conduction network. It achieves a prominent enhancement in thermal conductivity while maintaining excellent thermal energy storage capacity.

Battery thermal management tests demonstrated that PSAP could effectively reduce the surface temperature of batteries at 30 °C and 60 °C by 1.5 °C and 1.3 °C, respectively, indicating a more pronounced temperature-suppression effect than PAP. Under hot-plate heating at 80 °C, the surface temperature of PSAP was stably maintained at 52 °C, well below the heating temperature, indicating outstanding passive temperature regulation, heat dissipation, and thermal insulation performance. This oriented PNT@SiO_2_/cellulose aerogel phase change material exhibits excellent thermal conductivity, high phase change enthalpy, good dimensional stability, and outstanding thermal management performance, providing an efficient, reliable, and practical thermal management solution for power batteries.

## Figures and Tables

**Table 1:** Phase change properties of PEG, PAP, and PSAP.

Sample	Heating Process		Cooling Process	
	H_m_ (J/g)	T_m_ (°C)	H_c_ (J/g)	T_c_ (°C)
PEG	171.5	61.2	156.5	46.3
PAP	154.1	61.5	141.3	45.8
PSAP	158	61.1	149.7	45.7

**Table 2:** Performance comparison of the PSAP between this work and previous works.

Sample	Thermal Conductivity (W/m·K)	Latent Heat (J/g)	ΔT (°C)	Ref.
PEG-2.0SiC	0.393	167.3	3.6	[31]
SiHD/EG	1.83	116	2	[32]
PI@TD@GO5	0.14	140.9	3	[33]
MPCM/MXene	0.13	97.12	2.2	[34]
PSAP	0.93	158	1.5	This work

## Data Availability

The raw data supporting the conclusions of this article will be made available by the authors on request.

## Supplementary Materials

The following supporting information can be downloaded at https://www.mdpi.com/article/10.3390/nano16120709/s1, Figure S1: (a) weight of PSA and PSAP (b) weight of PA and PAP; Figure S2: Schematic diagram of battery charging and discharging temperature test device; Figure S3: Photos of PSAP samples and photos of PSAP wrapped on a 18,650 battery; Figure S4: Thickness of PSAP; Figure S5: Time-surface temperature image of 18,650 lithium battery at 2C charge–discharge rate; Table S1: Parameters setting of battery charge and discharge processing.

## Abbreviations

The following abbreviations are used in this manuscript:

PPy	Polypyrrole
PEG-8000	Polyethylene glycol 8000
CNFs	Microfibrillated cellulose nanofibers
PA	PNT aerogel
PSA	PNT@SiO_2_ aerogel
PS	PNT@SiO_2_
PAP	PNT aerogel/PEG
PSAP	PNT@SiO_2_ aerogel/PEG
PTFE	Polytetrafluoroethylene
PEI	Polyethyleneimine
CMC	Sodium carboxymethyl cellulose
TEM	Transmission electron microscope
SEM	Scanning electron microscope
EDS	Energy dispersive spectroscopy
FT-IR	Fourier transform infrared spectroscopy
DSC	Differential scanning calorimetry
XPS	X-ray photoelectron spectroscopy
SOC	State of charge
