# Supplementary material for: Preparation of PNT@SiO2 Aerogel Composite Phase Change Material with Oriented Structure and Its Thermal Management Characteristics for Battery
Source: Nanomaterials (Basel). 2026 Jun 9;16(12):709. doi: 10.3390/nano16120709 (PMC13305012; doi:10.3390/nano16120709)
Supplement: Supplementary file 1 [file nanomaterials-16-00709-s001.zip › nanomaterials-4344882-supplementary.pdf]

Preparation of PNT@SiO_2 Aerogel Composite Phase Change Material with Oriented Structure and Its Thermal Management Characteristics for Battery

Silong Wang, Wei Yan *, Pan Sun and Jun Yuan

School of Nuclear Science, Energy and Power Engineering, Shandong University, Jinan 250061, China; 202520783@mail.sdu.edu.cn (S.W.); 202334587@mail.sdu.edu.cn (P.S.); 202514604@mail.sdu.edu.cn (J.Y.)
* Correspondence: yanwei@sdu.edu.cn

Characterization testing technology and related instruments

The microstructures of the samples were obtained by SEM (JEOL JSM-7001F, Japan). A field emission SEM also obtained the energy dispersive spectroscopy (EDS) elemental mappings of samples. The powder samples of PNT and PS, and the block samples of PA and PSA, are uniformly stuck on the copper sample table through double-sided carbon conductive tape to ensure the conductive communication between the samples and the sample table. The samples were sprayed with gold to improve surface conductivity and facilitate observation of their micro-morphology.

The chemical structures of the PS, PA, PSA, PAP, and PSAP were analyzed by KBr sampling, Fourier transform infrared spectroscopy (FT-IR, whose type was Thermo Fisher Scientific Nicolet iS20, Waltham, MA, USA).

The element composition and chemical bond of composite phase change materials were analyzed by Thermo Scientific K-Alpha X-ray electron spectrometer in the United States and Mono Al$K\alpha$ source, Waltham, MA, USA.

The latent heat and temperature of phase transition were obtained by analyzing results measured from DSC (calorimeter model: Perkin Elmer DSC8000, Waltham, MA, USA). All the samples were tested in a nitrogen atmosphere with a flow rate of 20 mL/min, and in the temperature range of 30-100°C with a heating rate of 5 °C/min.

The thermal conductivity of the samples was measured using a Thermal conductivity meter (whose type was TC3000E, XIATECH, Xi'an, China). At least five measurements were taken for each sample to ensure the repeatability of the results.

The infrared images of the composite membranes were obtained by Fotric 326+ infrared thermal imager (FOTRIC - Shanghai Thermal Image Science & Technology Company, Shanghai China).

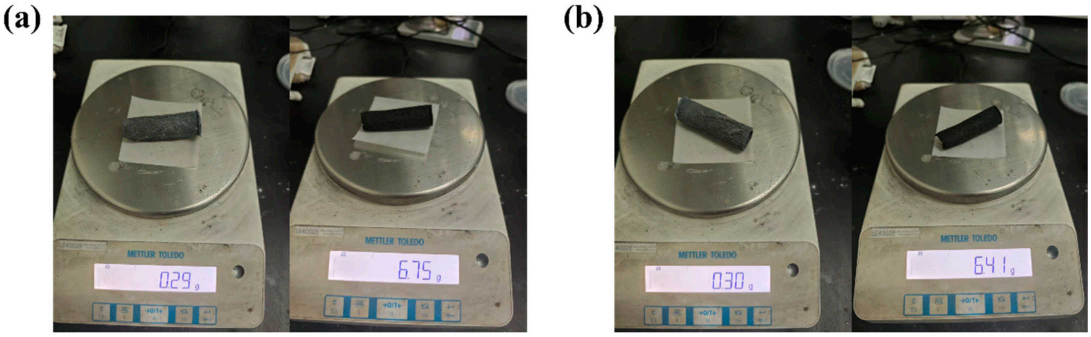

Figure S1. (a) weight of PSA and PSAP (b) weight of PA and PAP.

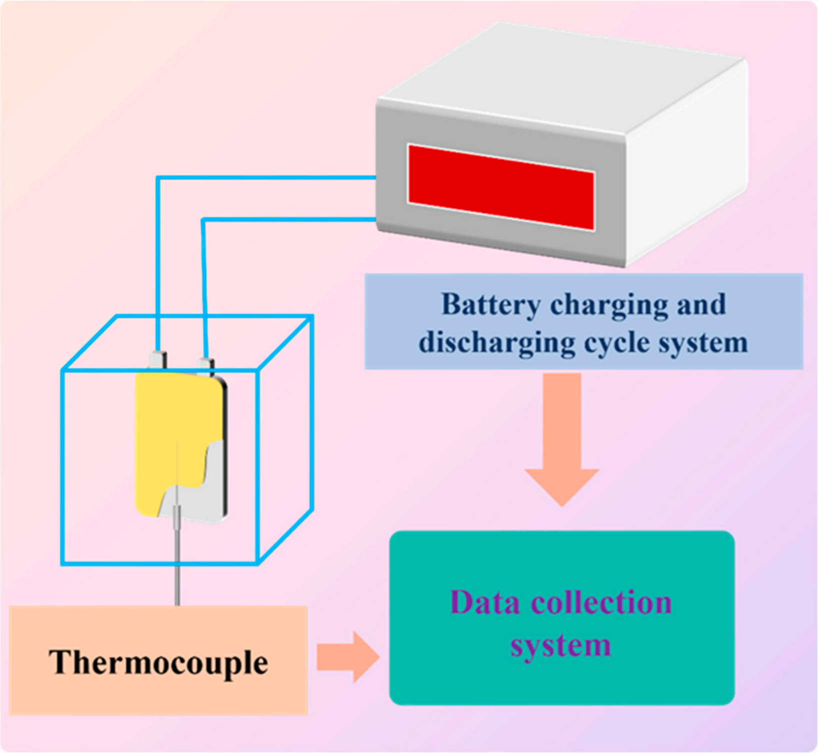

Figure S2. Schematic diagram of battery charging and discharging temperature test device.

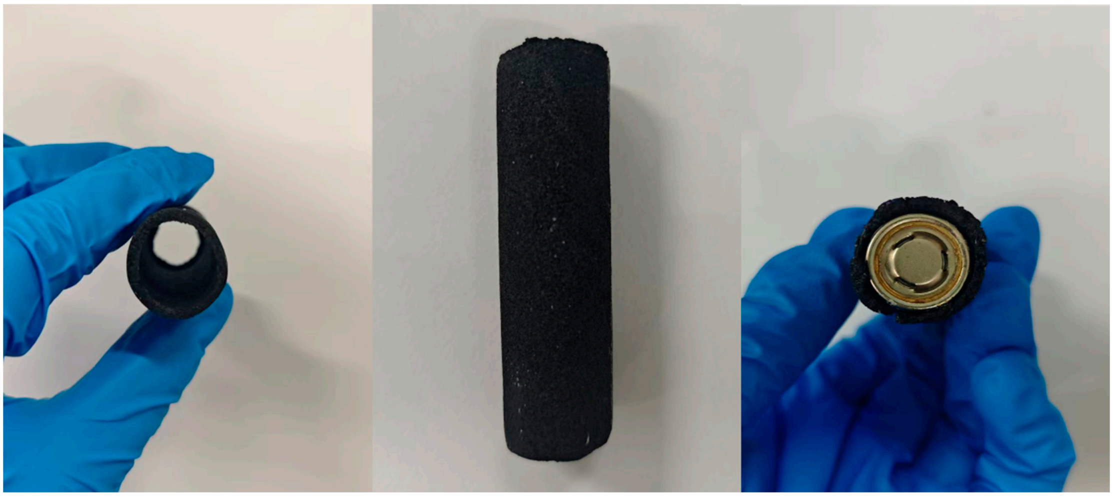

Figure S3. Photos of PSAP samples and photos of PSAP wrapped on a 18650 battery.

Table S1. Parameters setting of battery charge and discharge processing.

Procedures \ Parameters	Voltage (V)	Current (mA)
Constant current charging	4.2	4400
Constant voltage charging	4.2	100 (cutoff current)
Constant current discharging	4.2	4400
Standing	20 minutes	

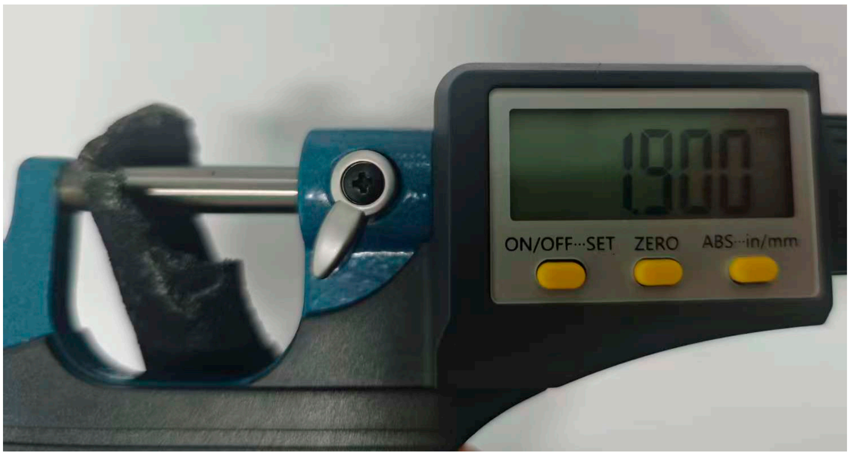

Figure S4. Thickness of PSAP.

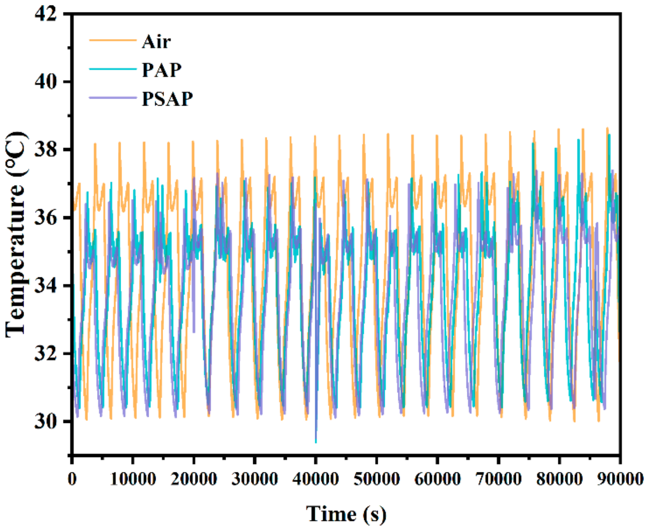

Figure S5. Time-surface temperature image of 18650 lithium battery at 2C charge-discharge rate.
